# Surgical Reconstruction with the Remnant Ligament Improves Joint Position Sense as well as Functional Ankle Instability: A 1-Year Follow-Up Study

**DOI:** 10.1155/2014/523902

**Published:** 2014-10-22

**Authors:** Kamizato Iwao, Deie Masataka, Fukuhara Kohei

**Affiliations:** ^1^Fukuhara Orthopedic Clinic, 4-4-8 Ujinanishi, Minami-ku, Hiroshima-shi 734-0014, Japan; ^2^Graduate School of Health Sciences, Hiroshima University, 1-2-3 Kasumi, Minami-ku, Hiroshima-shi 734-855, Japan

## Abstract

*Introduction*. Chronic functional instability—characterized by repeated ankle inversion sprains and a subjective sensation of instability—is one of the most common residual disabilities after an inversion sprain. However, whether surgical reconstruction improves sensorimotor control has not been reported to date. The purpose of this study was to assess functional improvement of chronic ankle instability after surgical reconstruction using the remnant ligament. *Materials and Methods*. We performed 10 cases in the intervention group and 20 healthy individuals as the control group. Before and after surgical reconstruction, we evaluated joint position sense and functional ankle instability by means of a questionnaire. *Results and Discussion*. There was a statistically significant difference between the control and intervention groups before surgical reconstruction. Three months after surgery in the intervention group, the joint position sense was significantly different from those found preoperatively. Before surgery, the mean score of functional ankle instability in the intervention group was almost twice as low. Three months after surgery, however, the score significantly increased. The results showed that surgical reconstruction using the remnant ligament was effective not only for improving mechanical retensioning but also for ameliorating joint position sense and functional ankle instability.

## 1. Introduction

Lateral ankle sprain is an extremely common injury in sporting activities, with rupture of the lateral ankle ligaments accounting for more than 85% of all ankle sprains [1]. The anterior talofibular ligament (ATFL) and calcaneofibular ligament (CFL) are most often injured, especially the former [1, 2]. Repeated ankle inversion sprains result in chronic ankle instability. Chronic ankle instability may be defined in 2 ways: mechanical and functional. Mechanical ankle instability is the objective measurement of instability. It is the motion beyond the physiologic range of motion. The anterior drawer translation and talar tilt angle are used to objectively document the degree of mechanical ankle instability. On the other hand, functional ankle instability was described by Freeman as the subjective unstable feeling or complaint of a giving-way sensation of the ankle joint, with the etiology involving proprioceptive disorders as a result of previous ankle injuries [3]. Freeman suggested that the nerve fibers in capsular structures and ligaments of the ankle subserve the proprioceptive response, which helps stabilize the foot and ankle. Not only mechanical disruption of articular structures following ankle sprain, but also the deficit of proprioception may have a profound effect on neuromuscular control. Several mechanoreceptors have been observed in lateral ankle anatomical components, including the lateral ligaments, capsule, and retinaculum [4, 5]. The aim of this study was to assess functional improvement in chronic ankle instability after surgical reconstruction using the remnant ligament. We hypothesized that surgical reconstruction using the remnant ligament should improve decreased mechanoreceptor activity. Here, we describe functional improvement in chronic ankle instability after surgical reconstruction using the remnant ligament.

## 2. Materials and Methods

### 2.1. Patients

The patients with a history of repeated unilateral ankle sprain participated in this study (intervention group). The patients were 5 men and 5 women, with an average age of 25 (range, 16–30) years. All patients had chronic lateral instability both mechanical and functional with symptoms of a giving-way sensation. All patients were undergoing conservative treatment including rehabilitation prior to surgical intervention (range, 3 months–1 year). The patients whom conditions have not improved participated in the study. All patients were diagnosed clinically as having a lateral ankle ligament injury according to a preoperative assessment tool such as stress radiography (Table 1). Twenty healthy individuals (10 men and 10 women) who did not have a history of ankle sprain or any ankle pain were enrolled as the control group. Their average age was 25 (range, 15–30) years. The study design was approved by the institutional review board of the Human Experimental and Ethics Committee in our clinic, and written informed consents were obtained from all patients or their relatives.

### 2.2. Surgical Intervention

**Figure 1:**
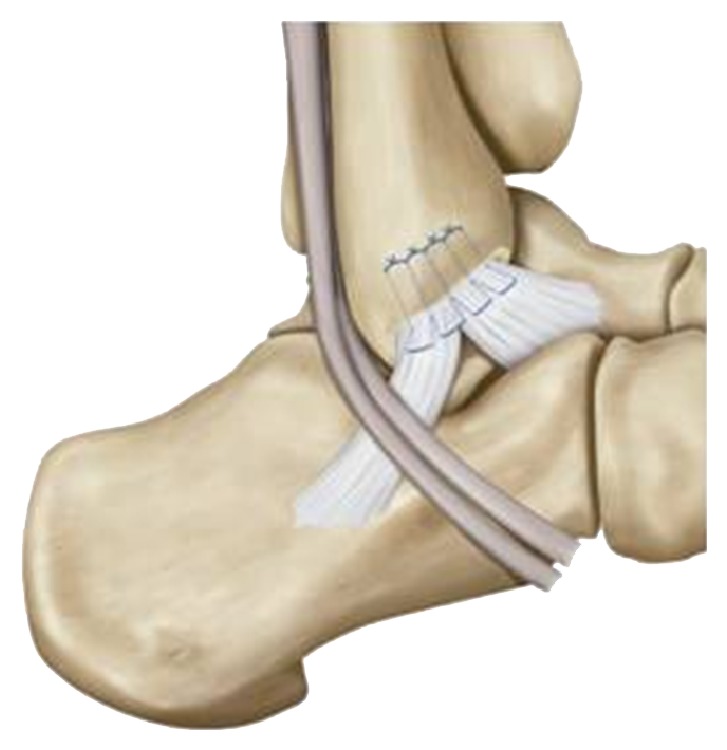
Our modified Broström method. Decortication was performed to improve the possibility of union between bone and ligament. And the remnant of the ATFL and CFL was sutured to their original attachment to the fibula through the “pull put technique” with the ankle in neutral position.

A modified Broström method was used for all patients with chronic ankle instability [6]. With the patient in a supine position, a short carved 5 cm incision was made over the ATFL. Subcutaneous dissection was carried down to the level of the capsule and joint of the anterolateral lesion of the ankle. In most cases, the remnant of the ATFL was confirmed by identification of the scar tissue adhered to the capsule and a thickened capsule. Then, the loosened CFL was seen from the tip of the fibula to its attachment on the calcaneus. The indication of this surgical procedure was based on the condition that the quality of the remnant of the ATFL and CFL, with 3–5 mm of thickness and 8–12 mm of width, was enough for the repair of the lateral ankle ligament, while the talar side of the ATFL and CFL attachment remained intact. The remnant of the ATFL, CFL, and capsule complex was completely detached from their attachment to the fibula, and decortication was performed to improve the possibility of union between bone and ligament. The remnant of the ATFL and CFL was sutured to their original attachment to the fibula through the pull put technique with the ankle in neutral position (Figure 1). After the tension of the repaired ligament and the ankle stability were checked, the wound was closed in layers.

The first measurement was performed on the day before operation in all patients. After surgery, all patients wore a cast for 4 weeks. After cast removal, physiotherapy was performed, which involved range of motion training and muscle training (Table 2). All patients received rehabilitation every day till one month postoperatively, and at least 3 times per week till three months postoperatively. In previous study, it is suggested that the reproduction of damaged lateral ankle ligament progresses at least 7 weeks [7]. So we conducted a follow-up assessment 3 months after surgery, when the operated ankle had regained similar range of motion to the healthy ankle, plantar flexion, dorsiflexion, inversion, and eversion. We additionally conducted follow-up assessments at the 6 months and 1 year after surgical treatment. The primary outcome measures were joint position sense and functional ankle instability, as described below.

### 2.3. Measurement of Joint Position Sense

**Figure 2:**
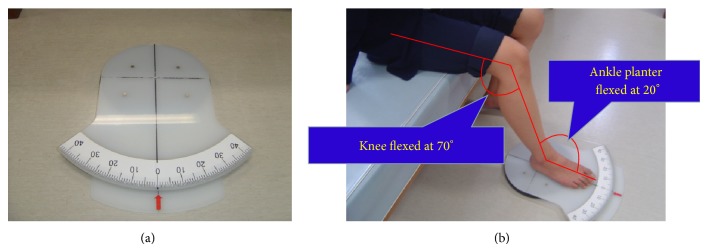
Goniometer footplate (a) and angles for the measurement of joint position sense (b).

The goniometer footplate (Nakamura Brace Co., Shimane, Japan; Figure 2) described by Nakasa et al. was used to assess joint position sense [8]. The subjects took off their shoes and socks. Then, they sat down with the knee flexed at 70°, one at a time, on the goniometer footplate at a plantar flexion angle of 20°. The goniometer footplate can rotate internally, which means that the axis of the foot movement is aligned with the axis of the ankle inversion movement. The center of rotation of the goniometer footplate is just below the tuberosity of the calcaneus. When the subjects moved their ankle to the index angle of inversion, they were asked to memorize the angle. Then, the ankle was returned to the 0° position. After that the subjects were blindfolded to eliminate visual input, and they moved their ankle actively to match the previous index angle. The index angle was decided using a table of random numbers to 1 of 6 positions (5°, 10°, 15°, 20°, 25°, 30°), always starting from 0°. The absolute difference between the index angle and replication angle was recorded as the joint position error. The absolute error of joint position sense was measured in triplicate. The mean of the 3 trials was used for analysis.

### 2.4. Evaluation of Functional Ankle Instability

We investigated subjective sensation of instability—the chief complaint in cases of functional ankle instability—by means of a questionnaire. We used a scoring list for the evaluation of functional ankle instability [2]. This scoring list is composed of 8 items: pain, swelling, sensation of instability, difficulty of motion, walking up and down stairs, running, activities of daily living (ADL), and need of some support. The total number of scoring point is 100, with levels divided into excellent (91–100 points); good (81–90 points); fair (61–80 points); and poor (<60 points). The subjects with less than 81 points were considered to have functional ankle instability.

### 2.5. Statistical Analysis

Statistical analysis was performed using SPSS version 6.xJ (SPSS Inc., Chicago, IL). We employed Dunnett's test for multiple comparison of the joint position sense error between the control and intervention groups. For comparison of the functional ankle instability score between the 2 groups, an unpaired *t*-test was used. A one-way analysis of variance (ANOVA) was performed, followed by paired sample *t*-test. A *P* value of less than 0.05 was considered to be statistically significant.

## 3. Results

### 3.1. Joint Position Sense

**Figure 3:**
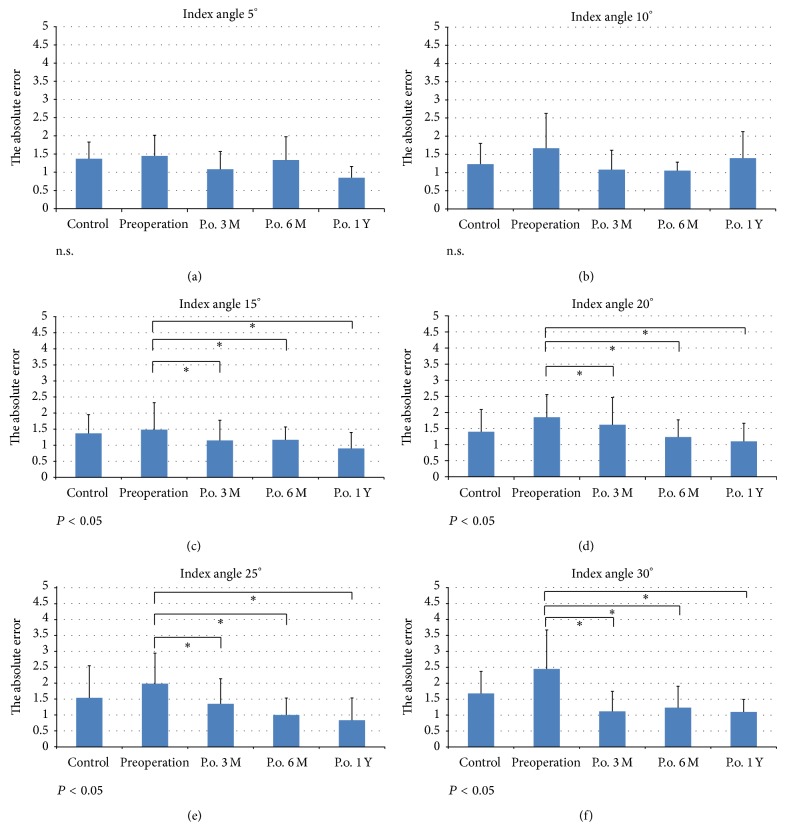
Joint position sense angles over the 1-year follow-up period. (a) and (b); there were no significant differences in the degree of error of joint position sense between control groups and unstable groups at index angle 5° and 10°. (c), (d), (e), (f); these values were significantly different from those found preoperatively for all index angles over 15° (*P* < 0.05). And there was no significant difference in the absolute error between 3 months, 6 months, and 1 year after surgery.

In the control group, the mean absolute error of joint position sense was 1.4 ± 0.5° at 5°, 1.2 ± 0.6° at 10°, 1.4 ± 0.6° at 15°, 1.4 ± 0.7° at 20°, 1.5 ± 1.0° at 25°, and 1.7 ± 0.7° at 30°. There were no significant differences in the degree of error of joint position sense between the dominant and nondominant ankle. There were also no differences between male and female patients. Therefore, we used the data of all 40 control ankles as a baseline for later comparisons. In the intervention group, the mean absolute error was 1.5 ± 0.6° at 5°, 1.7 ± 1.0° at 10°, 1.5 ± 0.8° at 15°, 1.9 ± 0.7° at 20°, 2.0 ± 1.0° at 25°, and 2.5 ± 1.2° at 30° before surgical reconstruction. There was a statistically significant difference between the control and intervention groups at all index angles over 15° before surgical reconstruction. Three months after surgical reconstruction in the intervention group, the mean absolute error of joint position sense was 1.1 ± 0.5° at 5°, 1.1 ± 0.5° at 10°, 1.2 ± 0.6° at 15°, 1.6 ± 0.9° at 20°, 1.4 ± 0.8° at 25°, and 1.1 ± 0.6° at 30°. These values were significantly different from those found preoperatively for all index angles over 15°. At six months after surgical reconstruction, the mean of absolute error was 1.3 ± 0.6° at 5°, 1.1 ± 0.2° at 10°, 1.2 ± 0.4° at 15°, 0.9 ± 0.5° at 20°, 1.0 ± 0.5° at 25°, and 1.2 ± 0.7° at 30°. One year after surgery, the mean of absolute error was 0.9 ± 0.3° at 5°, 1.4 ± 0.7° at 10°, 0.9 ± 0.5° at 15°, 1.1 ± 0.6° at 20°, 0.8 ± 0.7° at 25°, and 1.1 ± 0.4° at 30°. There was no significant difference in the absolute error between 3 months, 6 months, and 1 year after surgery (Figure 3).

### 3.2. Functional Ankle Instability

**Figure 4:**
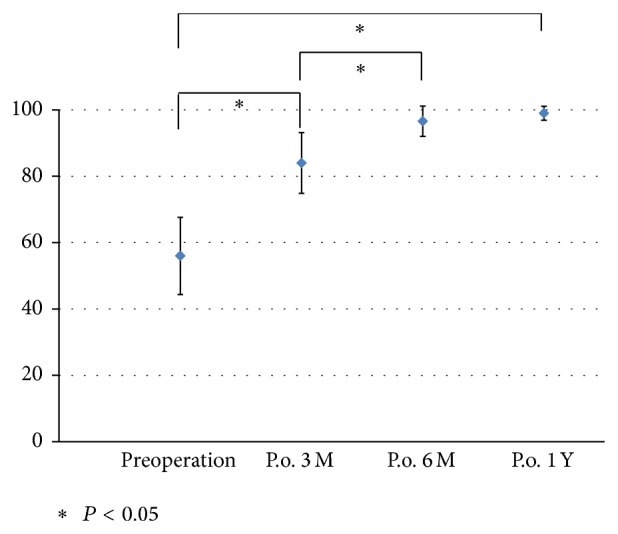
Functional ankle instability over the 1-year follow-up period. Three months after operative reconstruction, the score significantly increased (*P* < 0.05). The score increased further at 6 months and 1 year after operation.

In the control group, the mean score of functional ankle instability was maximum, 100 points. Before surgical reconstruction, the mean score of functional ankle instability in the intervention group was almost twice as low (56.0 ± 11.6 points). Three months after surgical reconstruction, however, the score significantly increased to 84.0 ± 9.1 points. The score increased further at six months and one year (96.6 ± 4.6 and 99.0 ± 2.1 points, resp.) after surgery. The difference between three and six months was also statistically significant (Figure 4).

## 4. Discussion

Adequate proprioceptive sensorimotor function of the ankle is a key factor in the treatment of ligament injury and chronic ankle instability [9]. Previous studies have reported significant differences in joint position sense between stable and unstable ankles [2, 8, 10, 11]. Indeed, in our study, the absolute error of joint position sense of the intervention (unstable) was significantly higher than in the control group. Importantly, we show that surgical reconstruction using the remnant ligament is efficient in improving ankle position sense and functional ankle instability in patients who had experienced an ankle sprain.

Halasi et al. reported that surgical treatment could improve the joint position sense of the unstable ankle [11]. This suggested that surgical reconstruction should be effective not only for mechanical ankle instability but also for functional ankle instability. In accordance with that study, Broström reported that 51 of 60 patients who underwent mid substance primary ligamentous repair reported minimal or no symptoms of instability at follow-up [6]. Now, using the remnant ligament, we also demonstrated excellent improvement of both joint position sense and functional ankle instability at three months after surgical reconstruction. Takebayashi et al. observed and mapped tension sensitive receptors in the lateral ankle ligaments [5]. Interestingly, the authors reported that the distribution of mechanoreceptor is not even in the lateral ankle ligament, and 93% of units were found either near the proximal or distal ends of the ligament, adjacent to the bone attachment. They also concluded that, from the viewpoint of the operative procedure, those who preserve the integrity of ligamentous detachments should be selected, as the density of the receptors is much greater in that area. Using the remnant ligaments in our operative treatment may be the reason for the excellent improvement of mechanoreceptor function and joint position sense, in addition to retensioning of the ligaments by surgical reconstruction. Surgical reconstruction using the remnant ligaments may be effective not only for retensioning of the lateral ankle ligaments but also for recovering the proprioceptive function. From the current study, it appeared that joint position sense was sufficiently improved at three months after surgery, as there were no differences between this and the later time points (six months and one year). In contrast, functional ankle instability continued to improve until six months after surgery. Hence, joint position sense was restored earlier than functional ankle instability.

These findings suggest that joint position sense could serve as an index to determine the appropriate time to start functional exercise for safe return to sports activity after ankle injury. Actually, functional ankle instability represents a loss of neuromuscular control, including proprioception, muscle weakness, muscle reaction time, and posture control [12]. If an appropriate rehabilitation program is offered to the patients, we may hasten improvement of functional ankle instability. Development of a rehabilitation program following ligament reconstruction is needed in the future.

The limitation of our study is that the number of patients was small, and surgical reconstruction using the remnant ligament was not compared with other ligament reconstruction techniques. A previous study has described that both primary and secondary repair yielded excellent or good results on perception of ankle stability [13]. Moreover, they concluded that each of those techniques yielded both good clinical and surgical outcomes. Further investigation with a larger number of patients and ligament reconstruction techniques would be required.

This study has 2 important results. First, before surgical reconstruction, the mean absolute error of joint position sense of the afflicted ankle was significantly larger than that of the healthy ankle. The scores of functional ankle instability of the intervention group were also significantly lower than those of the control group. This shows that proprioceptive malfunction has a role in the development of chronic ankle instability. Thus, deficit of joint position sense is a causative factor of functional ankle instability. Second, joint position sense with chronic ankle instability improved after surgical reconstruction using the remnant ligament. Using the mechanoreceptor-rich remnant ligament may have been the reason for the excellent improvement of joint position sense.

## 5. Conclusions

Based on these findings, we concluded that surgical reconstruction using the remnant ligament was effective not only for improving mechanical retensioning but also for ameliorating joint position sense and functional ankle instability. Joint position sense might be used in the future as a clinical assessment tool for determining the time to start functional exercise for safe return to sports after ankle injury.

## Figures and Tables

**Table 1:** Patient clinical data.

	Control (*n* = 20)	Intervention (*n* = 10)
Age: median (range)	24.5 (19–29)	27.6 (21–30)
Height (cm)	166.1 ± 7.3	165.5 ± 8.7
Weight (kg)	56.8 ± 6.6	55.6 ± 9.2
Male/female	10/10	5/5
Affected side (R/L)		5/5
Talar tilt angle (°)		9.7 ± 2.2
Anterior talar translation (mm)		6.0 ± 1.9

**Table 2:** Rehabilitation protocol after surgical intervention.

Surgery	p.o.2W		p.o.4W		p.o.2M	p.o.3M	
	Partial weight bearing exercise	→	Full weight bearing				
			Cast off				
			Range of motion exercise Muscle strength exercise (dorgi flexion, planter flexion)	→	Range of motion exercise Muscle strength exercise (inversion, eversion)		
					Balance disk exercise	$\vec{\;\;\;\;\;\;\;\;\;\;\;\;}\;$	
						Functional training (running, jump exercise, etc.)	→
